# What is the Role of Minimum Wages in Addressing Precarious Employment in the Informal and Formal Sectors? Findings from a Systematic Review

**DOI:** 10.1177/27551938241286463

**Published:** 2024-10-07

**Authors:** Carin Håkansta, Virginia Gunn, Bertina Kreshpaj, Nuria Matilla-Santander, David H. Wegman, Christer Hogstedt, Emilia F. Vignola, Carles Muntaner, Theo Bodin, Patricia O’Campo, Wayne Lewchuk

**Keywords:** Poverty, structural determinants of health, precarious employment, informal employment, income instability, policy initiatives

## Abstract

This article presents synthesized evidence from 16 studies examining initiatives with potential to mitigate workers’ exposure to precarious employment through the adoption of minimum wage policies. All studies were set in low-income countries and focused on both formal and informal workers. A systematic review of evaluated initiatives addressing precarious employment identified the evidence. We consider minimum wage policies as initiatives that could address precarious employment because of the central role of minimum wages in establishing employment terms for workers in precarious situations. We include initiatives aimed at formal and informal workers, given that precarious employment can exist in both sectors, that these workers share concerns regarding income and would benefit from minimum wage policies. The findings imply that minimum wage policies could increase precariously employed workers’ financial compensation, although with some differences and with little or no effect on employment security. It is not feasible to extend these conclusions beyond low-income economies due to differences with high-income economies in how the mechanisms through which minimum wage policies could impact worker compensation and employment security. However, they should serve as a reminder for high-income economies, many of which experience expanding informal sectors, about the need for related research and policy.

Income insecurity is often^1–3^ recognized as a dimension of precarious employment, while other researchers adopting a relational (social class) approach to precarious employment consider income insecurity implicit in contract insecurity.^4,5^ Therefore, introducing or raising the minimum wage could play a role in addressing precarious employment, since it is mostly defined with low wages.^4,6^ There is extensive international literature on the use of minimum wage policy to address poverty, including formal evaluations of the impact of such policies. However, these studies do not specifically discuss precarious employment, nor do they evaluate the impact of the policies on precarious employment. Instead, they focus on the employment effects of minimum wage policies^7^ and their effectiveness in raising the overall income from work of all working people, not only those in precarious employment, to some minimum level and the reduction of wage inequalities, including those between men and women.^8,9^

This article discusses the impact of minimum wage policies on workers in precarious employment and it focuses on two dimensions of precarious employment, income inadequacy and employment insecurity, using synthesized evidence identified through a systematic review of evaluated initiatives. We consider minimum wage policies as initiatives that could address precarious employment because minimum wages play a central role in establishing the terms of employment for workers in precarious situations. Since they are less likely to be union members, minimum wage policies allow workers in precarious employment to gain a degree of power over wages that they never had, or lost, due to the changed nature of contracts, security, and rights. An adequate minimum wage is an important part of fair terms of employment,^10^ as it protects workers in low-paying jobs such as many of those in precarious, including informal, employment arrangements.

The article is organized in five sections. The first section introduces key concepts such as minimum wages, precarious employment, and informal employment. It also reviews key arguments for and against the use of minimum wage policies, including arguments against their inclusion among initiatives that could be used to address precarious employment. The second section provides a brief description of the methods used to perform the systematic review. The third section synthesizes key findings, including study characteristics, and the fourth section contains a discussion of the findings. The concluding section addresses theoretical, practical, and policy implications.

## Key Concepts Covered: Minimum Wages, Precarious and Informal Employment

Minimum wages are defined as “the minimum amount of remuneration that an employer is required to pay wage earners for the work performed during a given period”^11^ and should aim at supplementing and strengthening other social and labor market policies, including collective bargaining, used to define conditions of employment and working conditions.^12^ Minimum wages are a policy tool used broadly across the globe, being implemented in over 90 percent of the International Labour Organization (ILO) member states.^13^ However, not all countries make use of minimum wage policies. For example, Sweden does not have a minimum wage but determines salary levels through collective bargaining.^14,15^

The history of minimum wage policy goes back to the late 19th and early twentieth century, when they were introduced in Australia, New Zealand, and the United Kingdom, for example, in response to precarious work in economic sectors such as boot making and tailoring, at the time typically referred to as *sweated labor*.^16^ Such initiatives continue today; for instance, the recent establishment of a minimum wage for harvest workers in Australia not only addressed their income inadequacy but offers some relief from the pressure to work faster and cut corners in an already hazard-prone sector.^17^

The precarious employment construct has no generally accepted definition, but public health scholars agree that it includes aspects such as employment insecurity, inadequate financial compensation, or income volatility, along with a lack of rights and protection in the employment relationship.^2–4,10^

According to the Organisation for Economic Co-operation and Development (OECD), informal employment refers to workers that neither in law nor practice are subject to income tax, national labor legislation, social protection, or the right to employment benefits.^18^ The level of informal sector employment is persistently high; in 2016 it was estimated to be more than 60 percent of global employment.^19^

The discussion about precarious employment is predominantly taking place in high-income nations. There, the share of standard forms of employment, typically understood as permanent, full-time, with a dependent relationship between the employer and the employee,^20^ used to be high but is now shrinking.^21–25^ The increase in nonstandard forms of employment, including self-employment, in high-income economies^20^ is significant to this shift from a dependent employee–employer relationship, especially since many protective policies, including minimum-wage policies, are built around standard employees, not self-employed workers.^26^ Precarious and informal employment are different concepts, and, in most instances, the concept of precarious employment is used within the context of formal employment. Nevertheless, scholars have compared the spread of precarious employment in high-income countries with the informal sectors of low-income countries, exemplified, for instance, by the similarities between informal sector entrepreneurs in low-wage economies and self-employed workers in high-income countries, pointing to a global “precariat”.^27^ Furthermore, researchers have highlighted the increasing insecurity of global employment relations, leading workers in all countries to transition between different employment arrangements, including formal, informal, and periods of unemployment,^28,29^ especially during periods of economic turmoil.^30,31^

The decision to include initiatives addressing precarious employment that focused on both formal and informal workers is based on several factors. First, key dimensions of precarious employment, such as employment insecurity, income inadequacy, or limited rights could exist in the formal as well as the informal sector. Second, since low wages could affect not only formal workers in precarious employment but also informal workers, whose income insecurity is implicit given their contract insecurity, both groups of workers could benefit from minimum wage policies. Third, there are many concerns shared between formal workers in precarious employment and informal workers regarding income, employment security, social protection, and other elements constituting decent work. Fourth, given that precarious employment is dependent on characteristics of the employment relationship,^2,32,33^ an argument could be made that all workers in the informal sector are in precarious employment since they lack a formal relationship with their employer. Fifth, because national labor and social protection systems are typically designed to address the standard employment relationship, workers in nonstandard forms of employment, who are often excluded from such systems, may be particularly exposed to various degrees of precarity and the risk of informality.^34^

## Brief Review of Arguments for and Against Minimum Wage Policies

Minimum wages are amongst the most broadly and consistently utilized labor market interventions across the world, irrespective of the level of economic development of countries using them.^35^ Minimum wage policies are adopted to protect workers against exploitative pay and support the redistribution of income from employers to workers^35,36^ in response to a general recognition that unregulated labor markets do not generally produce decent wages for workers.^35^

The literature on minimum wage policies features an intense debate between two groups. One argues that minimum wages protect the fair remuneration of employees and do not affect employment negatively.^37^ The other argues that minimum wages reduce employment and lead to increased wage inequality,^38^ despite cumulating evidence not supporting this claim and showing that minimum wages decrease wage inequalities.^37,39,40^ There is also criticism related to methodology, as the latter group neglects considering the independent role of institutional factors, such as declining unions and technological change on wage inequality.^38–40^

One argument in favor of minimum wage policies is their role in addressing poverty, thus contributing to general economic growth.^41^ Another is that minimum wages tend to reduce the range between the highest and lowest salaries, which speaks in favor of their positive impact on minimizing wage inequalities.^35,38^ In addition, according to the “lighthouse” hypothesis, minimum wage policies can have a spillover effect, working as a signal for wage bargaining, that also benefits the informal sector of the labor market.^41^ Overall, the arguments in favor of minimum wages focus on their positive impacts on workers, including the mitigation of disadvantages associated with employment precarity and informality.^38^ Additional benefits are reflected in various public health outcomes, such as improved mortality outcomes^42^ reduced smoking prevalence,^43^ improved infant and child health, and diminished financial anxiety.^44^ Attesting to the importance of and complex links between minimum wages and health, research on this topic continues to grow and diversify, expanding its focus to a range of other possible outcomes of minimum wages.^43^

Arguments against minimum wage policies often refer to classic labor theory, according to which the establishment of a wage floor higher than the equilibrium wage (i.e., where there is no excess labor supply) could lead to a reduction in the employment volume.^38^ However, there is a large body of evidence not supporting this claim.^37,39,40^ The critics suggest that minimum wage policies can lead to an increased share of informal employment or reduced employment as fewer employers will afford the additional expenses incurred by these policies, for example, higher staffing costs for same level of staffing.^9^ The switch from formal to informal employment or decreased employment rates can, in turn, mean that workers lose access to contracts, security, and rights previously enjoyed, which would then increase their employment insecurity.^18^ Research has shown contradictory results since the 1990s, and the debate between those in favor of and those against minimum wage policies has intensified. This debate is especially fierce in countries with segmented labor markets, in which workers with similar occupations and skills but different identity factors (e.g., race, gender, citizenship, or migration status) experience different employment and working conditions due to institutional^45^ and contextual factors.^46,47^ Examples of institutional factors are government regulations and trade union presence.^45^ Examples of contextual factors are the economic cycle in a country and the use of employer strategies that favor specific worker groups.^46,47^

While governments are the ones making decisions on whether to introduce a minimum wage policy, other important stakeholders are also involved in the minimum wage process. For example, organizations representing employers and formal workers, known as the social partners, often negotiate minimum wage levels. This creates a three-party involvement including the government, employers, and workers. Some countries do not have minimum wage policies at all. In Sweden, for example, lowest-level salary minimums are established in each industrial sector via bipartite collective bargaining between the social partners, thus excluding the government from the process.^48^ The argument used by both workers’ and employers’ organizations in Sweden for resisting centralized decision-making related to the setting of minimum wages is that it would obstruct the freedom of the social partners to impact wage negotiations.^14^ They worry that this could decrease the incentive for employers and workers to organize, leading to a reduction in collective agreement coverage and harming the Swedish labor relations model. Naturally, these considerations are crucial. However, this approach results in workers not covered by collective bargaining missing out on the direct benefits of minimum wage policies.^49,50^

Separate from the arguments against the use of minimum wage policies to address poverty and reduce wage inequality, there are arguments suggesting that such strategies are not adequate to address precarious employment. First, the construct of precarious employment shifts the analysis of work and health away from a narrow focus on income to a broader focus including employment relations and conditions, such as contracts, insecurity, or worker rights.^51^ According to this line of reasoning, minimum wage policies in isolation should not be used to address precarious employment since they only affect wages and no other key dimensions of precarity, such as contracts, rights, representation, and benefits. Low wage does not, by itself, constitute a precarious employment condition. Thus, focusing on minimum wage policies as potential strategies could take attention away from the goal of finding policies to reduce precarious employment. Second, seminal^51^ as well as contemporary^5^ research on precarious employment has excluded low wage as one of its dimensions because, otherwise, various categories of workers would not fit the criteria for precarious employment despite high levels of temporality and lack of benefits. The inclusion of income in the definition of precarious employment is specifically contested because it may lead to an undercounting of precariously employed workers by excluding those who suffer from job insecurity and a lack of benefits and rights but have a median or above median income (e.g., contract, seasonal faculty, or agency health workers). For this reason, several researchers incorporate this argument in their operationalization of the concept of precarious employment—they separate in their analyses low wages from the employment conditions most unique to precarious employment.^5^ Last, but not least, the effect of minimum wages on precarious employment is ambiguous and might depend on contextual labor market configurations.^7^ Some researchers argue that an increase in minimum wages makes low wage employment more attractive than unemployment, causing some workers to engage in paid, albeit low-wage and likely precarious, employment.^52^

Because income inadequacy constitutes a key dimension of precarious employment,^2,53,54^ and precarious employment is mostly defined with low wages,^4,6^ we consider minimum wage policies as suitable strategies to address precarious employment. Further, while we acknowledge that minimum wage policies do not purposely address workers’ employment arrangements, representation, or nonmonetary rights and benefits, we argue that they have the potential to reduce or mitigate income inadequacy through increased financial compensation, an important consideration given the overall scarcity of evaluated initiatives addressing precarious employment.^31^ Additionally, given recent evidence on the positive relationship between minimum wages and worker health in general,^43,44^ it is likely that such relation will also hold in precariously employed workers.

## Aim of This Article

The specific objective of this article is to discuss findings from 16 studies focused on evaluated initiatives with potential to eliminate, reduce, or mitigate workers’ exposure to precarious employment through the adoption of minimum wage policies. This article builds upon the aims of a larger review where the objectives were to identify, appraise, and synthesize existing research about the effectiveness of initiatives addressing precarious employment and its effects on the health and well-being of workers and their families.^55^

## Material and Methods

The review was guided by the 2020 Preferred Reporting Items for Systematic Reviews and Meta-Analyses (PRISMA) framework.^56^ We used the PRISMA guidelines to develop research questions and a systematic search process, to identify and select relevant studies, and to design processes of data extraction, data analysis, and data synthesis. A more detailed description of the methods used can be found in the review protocol^55^ and its PROSPERO registration.^57^

While this article only presents the outcome of the minimum wage publications included, the following description summarizes the approach used to conduct the complete systematic review.

Inclusion criteria:

1. Population of interest: workers (18 years of age and older, irrespective of gender, race, ethnicity, and migration status) and workers’ immediate or extended families.
2. Initiatives examined: initiatives that were purposefully designed to address precarious employment or that were designed for other purposes but that had the potential to address precarious employment and/or its effects on the health and well-being of workers and their families. Initiatives had to be both implemented and evaluated and were considered regardless of the evaluation results (successful, unsuccessful, or inconclusive). They were defined as broadly as possible and included interventions, policies, legislation/regulations, programs, guidelines, recommendations, collective agreements, and institutional practices.
3. Outcomes evaluated: focused on changes in prevalence of precarious employment, workers’ exposure to precarious employment, or the health and well-being of precariously employed workers and their families.
4. Study design: qualitative, quantitative, or mixed-methods study designs and evaluations.
5. Publication year and language: studies published from January 2000 to May 2021, in any language spoken by members of our review team: Catalan, Danish, Dutch, English, French, Italian, Norwegian, Romanian, Spanish, and Swedish.

Exclusion criteria:

1. Editorial, commentary, discussion paper, review.
2. No clear initiative implemented.
3. We excluded initiatives not evaluated formally or assessed using empirical data or initiatives with an evaluation that does not include a clear focus on reduction in precarious employment and/or on precarious, including informal, workers and/or their families, as well as publications that were not in a language spoken by members of the team. We also excluded initiatives designed to: facilitate or increase exposure to precarious employment; improve workers’ health through individual behavioral change without a focus on precarious employment; improve work performance or health, safety, or well-being of workers with disabilities without a focus on precarious employment; eliminate or reduce workers’ exposure to unemployment; eliminate, reduce, or mitigate the effects of unemployment on health and well-being; promote workers’ return to work after illness or injury without addressing precarious employment.

**Figure 1.**
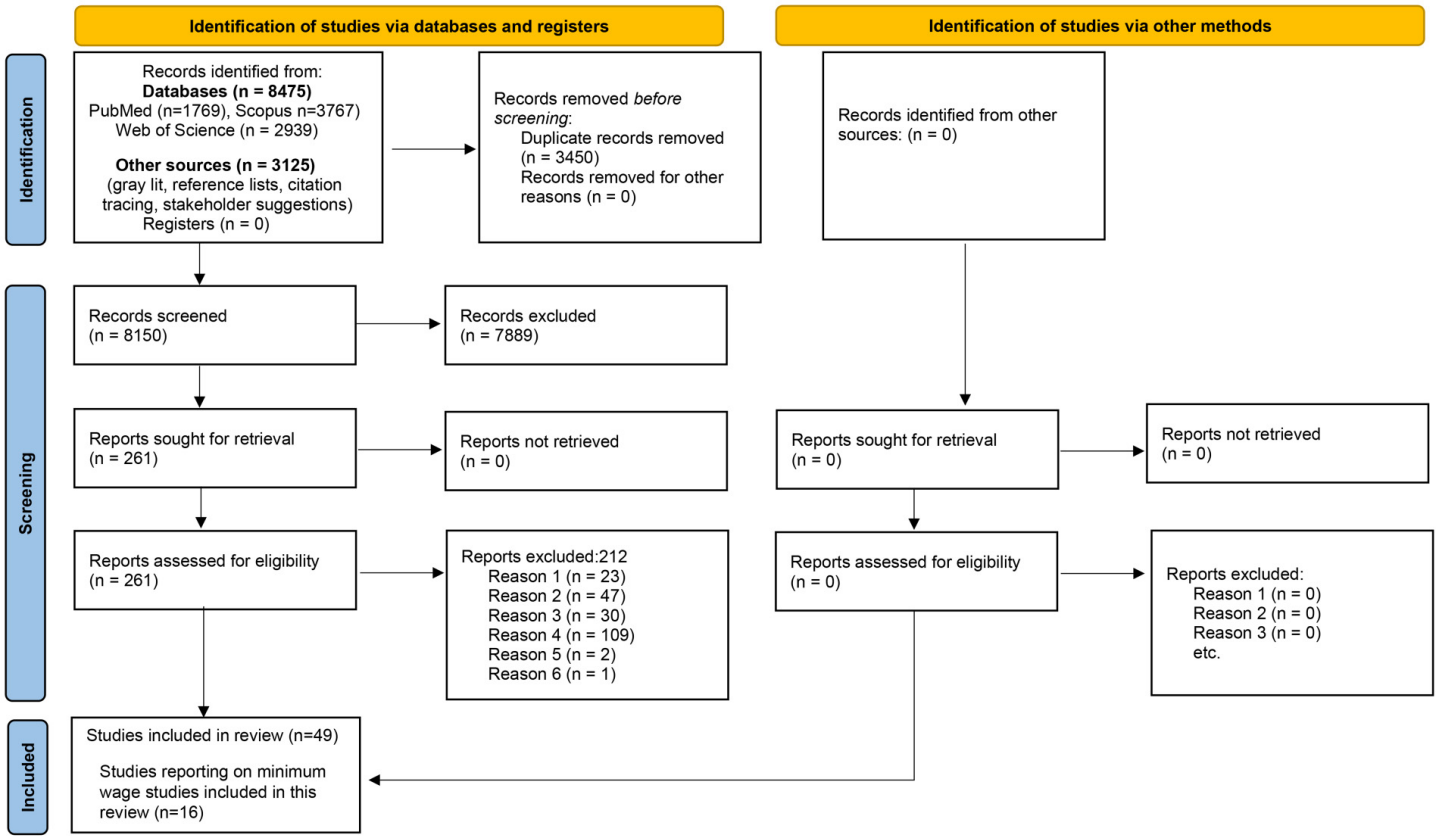
PRISMA 2020 flow diagram. Reasons: 1. Editorial, Commentary, Discussion Paper, Review; 2. No clear initiative implemented; 3. Initiative designed to facilitate precarious employment (PE) or increase exposure to PE; Improve workers’ health through individual behavioral change without a focus on PE; Improve work performance or health, safety, or well-being of workers with disabilities without a focus on PE; Eliminate or reduce workers’ exposure to unemployment; Eliminate, reduce, or mitigate the effects of unemployment on health and well-being; or Promote workers’ return to work after illness or injury without addressing PE; 4. Not evaluated formally or assessed using empirical data or The evaluation does not include a clear focus on the reduction of precarious employment and/or on precarious workers and/or their families. 5. Duplicate. 6. Not in a language mentioned in the protocol.

Our search strategies covered three academic databases (PubMed, Scopus, and Web of Science Core Collection) and three sources of grey literature (International Labour Organization, European Foundation for the Improvement of Living and Working Conditions, Centers for Disease Control and Prevention Community Guide of Evidence-Based Findings). We also consulted stakeholders for suggestions, reviewed the reference lists of included studies, and conducted forward citation tracing. The PRISMA flow diagram (see Figure 1) provides an overview of the selection process.

Publications that met all inclusion and none of the exclusion criteria were selected for the data extraction phase. Nine coauthors were involved in the screening and data extraction stages, consisting of a review of titles and abstracts against the inclusion criteria, and the full-text review, in which two independent reviewers confirmed if the publication fit the inclusion criteria. Disagreements were solved through discussions within the team. Included studies subsequently underwent a data extraction process in which two reviewers independently completed a tailored data extraction form providing all relevant information about the studies. All of the included studies were set in low-income countries, and they all address both formal and informal employment.

To assess the methodological quality of the studies, we opted for the Mixed Methods Appraisal Tool (MMAT).^58^ The tailored assessment questions and selection algorithm of this tool facilitates a clear distinction between quantitative study designs and make it suitable for various study methods. For each selected publication, two reviewers used the tool independently. Disagreements were solved through discussions.

To ensure that readers are well informed of key limitations of the studies included in the review and take them into consideration while making sense of the findings, we included a section dedicated to methodological considerations in Table 3, following the quality appraisal rating. This section draws attention to a range of study limitations, including data sources used, types of analyses conducted, timing of evaluations, sample composition, and attrition rates.

## Results

### Included Publications

The initial search produced 11,600 potentially relevant publications, of which 8,475 came from the academic databases and 3,125 from other sources (see Figure 1). We removed 3,450 duplicate entries, leaving us with 8,150 publications for the screening of titles and abstracts. After having selected 261 publications for full-text review, 212 were excluded as they met one or more of the exclusion criteria. Data extraction was subsequently done for the 67 remaining studies and during another confirmatory step, another 18 studies were excluded. The final systematic review thus comprises 49 publications, of which 16 focus on minimum wage policies and are discussed in this paper. In a previous publication by the authors, the results from 11 articles focusing on health and well-being are summarized.^59^ A separate manuscript summarizes the remaining 22 studies that focused on initiatives other than those related to minimum wage.^60^

### Characteristics of the Included Studies

**Figure 2.**
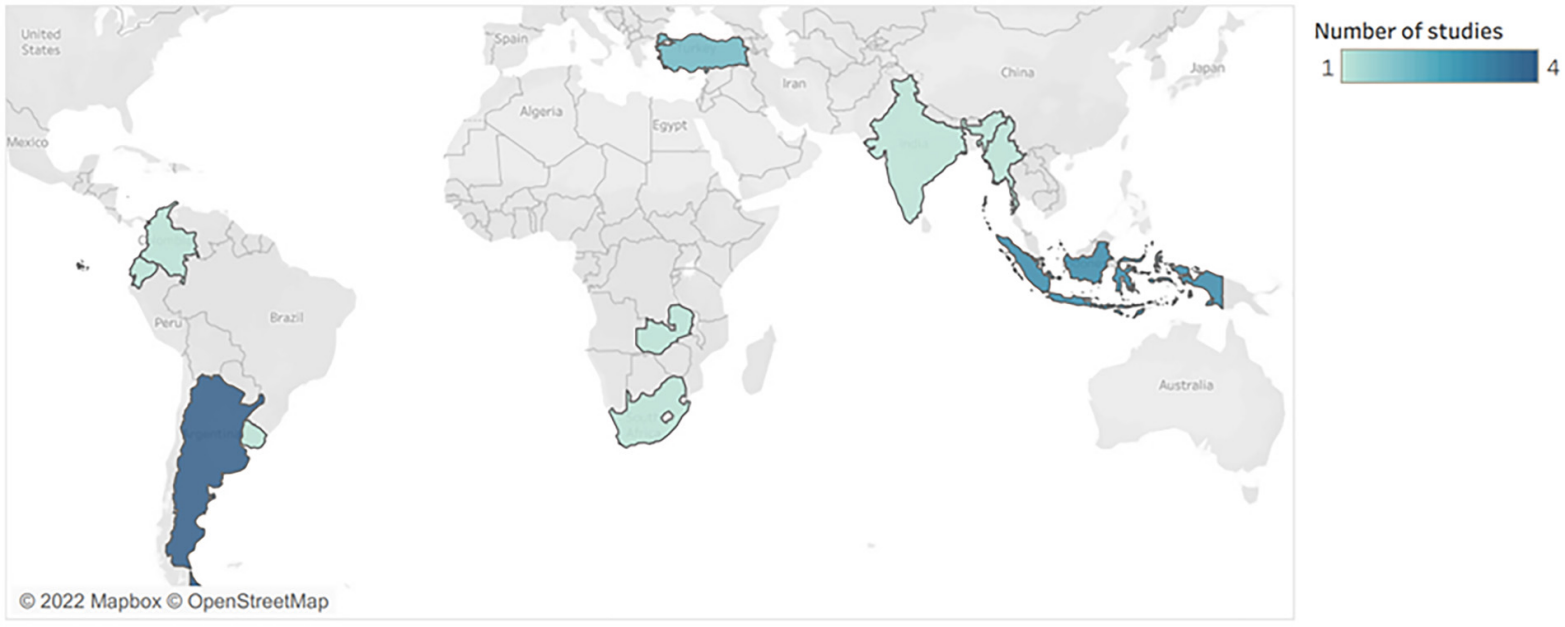
Geographical distribution of studies.

Table 1 presents an overview of several characteristics of the 16 studies. Applying the six regions of the United Nations’ geoscheme, the geographical locations of the studies were: Europe/Asia (two studies), Asia (five studies), Latin America (seven studies), Africa (two studies). A map in Figure 2 displays this geographical distribution of the studies. It should be noted that despite most minimum wage studies being focused on medium or high-income nations, the ones we found, which evaluated the impact of minimum wage policies on dimensions of precarious employment, all concerned lower-income nations.

**Table 1.** Characteristics of the Included Articles.

		Number of articles
Studies included		16
Regions represented by the countries examined	Africa	2
	Asia	5
	Latin America	7
	Europe and Asia (Turkey)	2
Study design*	Quantitative descriptive studies	15
	Mixed methods studies	1
Targeted economic sector°	Agriculture, forestry	2
	Domestic	5
	Retail	1
	Security	2
	Taxi	1
	No specific economic sector specified	11
Outcomes evaluated°	Financial compensation	12
	Employment security	14
Quality appraisal rating**	Low quality (0 to 2)	0
	Medium quality (3 to 5)	2
	High quality (6 to 7)	14

*This categorization of study design uses the categories included in the Mixed Methods Appraisal Tool (MMAT),2018 version.

**Quality appraisal rating interpretation: To calculate the rating, we used the number of “Yes” responses to the quality

assessment questions included in the MMAT 2018 version, including the two screening questions.

Low quality (0–2 “Yes” answers), Medium quality (3–5 “Yes” answers), and High quality (6–7 “Yes” answers).

°The sum can be more than 16 because several studies targeted several economic sectors and could have evaluated more than one outcome.

The study design for 15 of the 16 studies was quantitative descriptive. The other utilized mixed methods. Eleven studies did not focus on a specific economic sector, including one that focused on public and private enterprises, and five examined the effects of minimum wages on workers in one or more sectors recognized for their high levels of precarious employment such as domestic work (five studies), retail (one study), agriculture and forestry (two studies), security (two studies), and taxi (one study).

All studies evaluated the impact of minimum wage policies on workers’ compensation and/or their employment security, as illustrated by their movement across different types of employment arrangements, as detailed next. Specifically, 12 studies examined outcomes related to financial compensation and 14 examined outcomes related to employment security for either all formal sector workers, including those precariously employed, both formal and informal workers, or informal workers only, as detailed next.

The results from our quality appraisal rating show a body of high-quality research overall, with 14 studies deemed as high-quality and two as moderate quality.

Table 2 presents further details on the included studies. Except for one study published in 2004, all studies were published between 2013 and 2021. The language of 14 studies was English, one was written in French and one in Spanish. Three of the studies were book chapters and 13 were academic journal articles.

**Table 2.** Countries Examined, Language, Type of Evidence, Population of Interest, Targeted Economic Sector, and Study Objectives.

Study author(s); publication year	Countries examined/language	Type of evidence	Population of interest and targeted economic sector	Design and data collection approaches used to evaluate initiatives	Study objectives
Maloney and Mendez (2004)	Latin America, with Colombia as a case/English	Book chapter published by National Bureau of Economic Research	Formal and informal workers (no specific sector).	Quantitative descriptive study based on panel data from the National Housing Survey in Colombia since 1997 with a rotating sample of 25% of households. Uses numerical measures and kernel density plots to show the impact of minimum wage on the distribution of wages, with a focus on the informal sector and on higher wages. Tracks employment movement from having a job to becoming unemployed from one quarter to the next and not longer-term effects beyond three months.	To quantify the effects, at 3 months of raising the minimum wage on worker wages, and the move from employment to unemployment.
Bhorat et al. (2013)	South Africa/English	Academic journal article	Formal workers in sectors with high levels of precarious employment (retail, domestic work, forestry, security, and taxi).	Quantitative descriptive study based on 15 waves of biannual Labour Force Survey data (2000–2007). Using a quasi-experimental approach, two alternative specifications of a difference-in-differences model were applied in order to estimate the impact of wage laws on employment, wages, and hours of work in five sectors. A unique control group was established for each sector.	To assess the effects of minimum wage laws on employment, wages, and hours of work in five sectors.
Khamis (2013)	Argentina/English	Academic journal article	Formal and informal workers (no specific sector).	Quantitative descriptive study based on Argentine National Household data 1993–2004. As a control group, the paper exploits the different prevalence of low wage workers in regions of Argentina and assumes that regions with few low wage workers will be less affected by changes in minimum wage and can be used as the control.	To assess the effects of minimum wage on wages.
Groisman (2014)	Latin America, with Argentina as a case/English	Book chapter published by International Labour Organization	Formal and informal workers (no specific sector).	Quantitative descriptive study based mainly on pooled panel data (14,000 households) 2004–2013 and a simple distribution of minimum wage workers. It uses multinomial logistic regression to assess the effects of minimum wage on employment, informality and wages.	To assess the effects of minimum wage on employment, informality, and wages.
Aslan and Kirat (2015)	Turkey/French	Academic journal article	Formal and informal workers (no specific sector).	Quantitative descriptive study based on aggregate data from 26 annual observations collected by the Turkish National Statistics Institute (1988–2013). Regresses level of employment on minimum wage, level of economic activity, level of active population.	To assess the effects of minimum wage on wages.
Hohberg and Lay (2015)	Indonesia/English	Academic journal article	Formal and informal workers (no specific sector).	Quantitative descriptive study based on data on the self-employed (48,030 observations and 18,825 individuals) in Indonesia 1997–2007 from Indonesian Family Life Survey, three waves. The unit of analysis is the individual. Fixed effects regression at the individual level to assess the effects of minimum wage on informal and formal sector wages and employment.	To assess the effects of minimum wage on employment, informality, and wages.
Groisman (2016)	Argentina/English	Book chapter in Research on Economic Inequality	Formal and informal workers (no specific sector).	Quantitative descriptive study based on longitudinal, individual annual panel data (2004–2013) from Argentina National Institute of Statistics Permanent Household Survey, using multinomial logistic regression models. The analysis examines if people earning near the minimum wage lose jobs when the minimum wage changes.	To assess the effects of minimum wage and income transfer programs on the economic participation of the population and the informal sector.
Pratomo (2016)	Indonesia/English	Academic journal article	Formal and informal workers (youth) (no specific sector).	Quantitative descriptive study based on the National Labour Force Survey (2010–2012); a logit model is used to examine the distribution of youth employment across five different categories of the employment relationship when the minimum wage changes. The unit of analysis is the individual. The model includes separate analyses for urban and rural areas and for gender.	To assess the effects of minimum wage on youth with different employment status 2010–2012.
Jimenez (2018)	Argentina/Spanish	Academic journal article	Formal and informal workers (no specific sector).	Quantitative descriptive study based on data from the Argentine Permanent Household Survey for the period 2004–2005, a difference in difference approach is used to examine a) regional variety in prevalence of workers earning near or below the minimum wage and b) median wage within urban regions of Argentina.	To assess the effects of a minimum wage increase in 2004–2005 on transitions to quality jobs.
Wong (2019)	Ecuador/English	Academic journal article	Formal low-income workers including workers in sectors with high levels of precarious employment (domestic work and agriculture).	Quantitative descriptive study based on individual panel data drawn from a household panel in 2012 (National Institute of Statistics and Census), the difference in difference approach was used to analyze the effects on hours worked and wages among low-wage workers of the 2012 minimum wage increase.	To assess the effects of a minimum wage increase on wages and hours worked among low-wage workers in Ecuador.
Gudibande and Arun (2020)	India/English	Academic journal article	Informal workers (including maid servants, cooks and watch men).	Quantitative descriptive study based on cross-sectional household data from a National Sample Survey in India (2004–2012). Units of analysis are the state and the individual in different states. Exploits the fact that not all states have a minimum wage for domestic workers. Uses matched pairs in treatment/nontreatment states (in total 16).	To assess the impact of minimum wage legislation on wages and employment opportunities.
Işlk et al. (2020)	Turkey/English	Academic journal article	Formal and informal workers (no specific sector).	Quantitative descriptive study based on data from the annual Household Labor Force Survey (2009–2016). Unit analysis is aggregate data on key sectors of the workforce (age, education, formal, informal). Individual data is used to assess outcomes by controlling for the % of workers in a region affected by the change in minimum wage. Uses simple before and after comparisons of prevalence of workers below or near minimum wage and regression analysis to study regional effects of a national minimum wage.	To assess the effects of a minimum wage increase on wages, informality, and employment.
Nyirenda and Chibomba (2020)	Zambia, with the City of Chipata as a case/English	Academic journal article	Formal workers in sectors with high levels of precarious employment (domestic work).	Mixed methods study based on a descriptive research design and purposive sampling, using researcher collected surveys and interviews with employers and domestic workers in 30 households in 2018. It also uses secondary data from textbooks, journals and other online resources. A combination of thematic and statistical analyses was used.	To assess the effects of minimum wage policy on wages and employment opportunities.
Siregar (2020)	Indonesia/English	Academic journal article	Formal and informal workers (no specific sector).	Quantitative descriptive study based on aggregate panel data covering 26 provinces in Indonesia, collected from the Indonesia Labor Force Survey (2001–2015) and the Ministry of Manpower and Transmigration. It uses a dynamic panel data estimation method, specifically the general methods of moments estimator to correct for error term problems.	To assess the impact of minimum wage on employment, formal and informal employment.
Katzkowicz et al. (2021)	Uruguay/English	Academic journal article	Formal and informal workers (domestic work).	Quantitative descriptive study based on cross sectional pooled data from National Household Survey in Uruguay (2006–2016) using a dual-economy density-discontinuity design with latent values that would have existed without a minimum wage policy. Estimating the impact of minimum wages on unemployment, wages, and mobility of women domestic workers between the formal and informal sectors.	To assess the impact of minimum wage on wages, unemployment, and formal-informal sector mobility among women.
Kyaw and Cho (2021)	Myanmar/English	Academic journal article	Formal and informal workers (no specific sector).	Quantitative descriptive study based on panel data from the World Bank Enterprise Survey across five major industrial sectors (in 2014 and 2016) using fixed-effects regression to estimate whether enterprises substitute machines for labor due to the rise of the minimum wage.	To assess the effects of minimum wage policy on full-time and part-time employment.

The population of interest varied but for most studies (12 of them) both formal and informal workers were the target. We assume that the formal, low-wage workers included in these studies were likely to have been precariously employed. One study focused on informal workers only and the remaining three on formal workers in sectors known for their high levels of precarious employment such as domestic work, retail, agriculture, or forestry, who we also assume were likely to have been precariously employed.

Data collection approaches relied on data provided by national statistical organizations along with surveys and interviews carried out by the researchers in combination with secondary data. The study unit of analysis included individual, firm, region, or socioeconomic groups. Most studies used simple regression analysis to explore effects before and after changes in the minimum wage or among sectors that adopted or not the policy. Only several studies considered or accounted for context or broader changes occurring in the labor market that could affect resulting changes in minimum wages or other outcomes.

All studies had a macro-level focus, on country- or sector-level minimum-wage policies. The overall objectives across all studies were to examine the effects of minimum wage policies on workers’ financial compensation and/or employment security, as detailed next.

### Implemented Initiatives, Evaluated Outcomes, Results, and Methodological Considerations

Table 3 provides an overview of the implemented initiatives covered in each study, the outcomes evaluated, the results, and our overall appraisal rating for each study along with several methodological considerations to support the interpretation of findings. These considerations provide additional information about the study design, often related to limited access to data.

**Table 3.** Implemented Initiatives; Evaluated Outcomes; Results; Quality Appraisal Rating, Methodological Considerations.

Study author(s); year of publication	Implemented initiatives	Outcomes evaluated		Results	Quality appraisal rating*	Methodological considerations to support the interpretation of results
		Financial Compensation	Employment Security			
Maloney and Mendez (2004)	Two increases in minimum wages in Colombia between 1997 and 1999.	✓	✓	The rise in minimum wage increased the wages of workers earning close to the minimum wage, including those in the informal sector, as well as the probability of becoming unemployed. In both cases the effect was smaller for higher income earners.	High	A limitation of the study is that it only tracks employment movement from having a job to becoming unemployed. It does not study the reverse from unemployment to employment. It also only tracks changes from one quarter to the next, without focusing on longer term effects beyond three months.
Bhorat et al. (2013)	Increases in minimum wages in different sectors of the South African economy, first introduced in 1999.	✓	✓	No significant decline in employment observed. Wage increases in three of the sectors (retail, domestic, and security) sufficient to outweigh intensive margin adjustments so that workers experienced an improvement in their real monthly income. For the remaining two sectors (forestry and taxi), workers may not have been any better or worse off in real income terms.	High	Given spillover effects affecting the control group, the impact estimates on employment and wages may be biased upwards (e.g., workers in the control groups may experience a negative effect on wages due to workers in sectors impacted by minimum wage policies losing their jobs and finding employment in other sectors).
Khamis (2013)	Increases in minimum wages in Argentina between 1993 and 2004.	✓		Finds that an increase in the minimum wage leads to raised wages in the informal sector but not in the formal sector. These findings suggest that the minimum wage may act as a reference wage for the informal sector and this sector's noncompliance with certain labor laws does not automatically translate into noncompliance with other labor laws, including minimum wage.	High	Given limited data availability, only short-term effects are examined.
Groisman (2014)	Increases in minimum wages in Argentina between 2003 and 2011.	✓	✓	Finds that minimum wage results in increased wages, no decrease in employment and no significant increase in informal employment or unemployment. The salary gap between workers in formal and informal employment did not change.	High	Because pooled data was used, there was no correction for growth in the economy etc. over time. The analysis covers only short-term effects in the one year following each minimum wage modification, thus making the understanding of longer-term effects impossible.
Aslan and Kirat (2015)	Existing minimum wage policy in Turkey, first introduced in 1936.	✓	✓	Indicates that an increase in minimum wage reduces employment, but by less than the increase in wage levels, resulting in an increase in GDP. In the long run, a minimum wage increases formal employment and reduces informal employment.	High	The analysis considers the different effects on female and male workers.
Hohberg and Lay (2015)	Existing minimum wage policy in Indonesia, first introduced in 1956.	✓	✓	Finds that the adoption of minimum wage legislation has significant positive effect on formal sector wages but no spillover effects on informal workers. While there was no impact on total employment. employment increased in the formal sector and decreased in the informal sector. In addition, unemployment increased.	High	Low attrition rate across the three waves. The informal worker definition different than that of other studies, classifying private or government workers as formal and the workers who report “self-employed” or “unpaid family worker” as informal given that they are not legally covered by minimum wage policies.
Groisman (2016)	Increases in minimum wages in Argentina 24 times between 2002 and 2014.	✓	✓	The change in minimum wage did not negatively affect employment and only a very small number of formal workers moved to informal employment. There was no change in the salary gap between formal and informal workers.	High	Compares four categories: formal salaried; informal salaried; not occupied; occupied non-salaried, however, without clearly outlining the difference between informal salaried and occupied non-salaried.
Pratomo (2016)	Increases in minimum wages in Indonesia in 2010 at national level (with differences among provinces and districts).		✓	Concludes that a raise of the minimum wage reduces the probability of youth mobility from informal to formal employment. The effects of increased unemployment strongest among youth with more education, suggesting they can wait for a good job when the labor market is disturbed. The change had less effect on young women than men and less impact in rural than urban areas.	High	Low attrition rate for the second-year analysis. The implementation of the minimum wage policy varied across the provinces and sectors analyzed.
Jimenez (2018)	Increases in minimum wages in Argentina in 2004–2005.		✓	Finds that increases in minimum wage reduces overall employment and reduces the prevalence of workers in the informal sector moving to better quality jobs.	High	Limited to medium and large enterprises in the public sector. Relies on regional variances in prevalence of workers earning near or below minimum wage and the median wage within urban regions. Provides relevant details on job quality including job satisfaction, job stability, and extension of the working day: over-employment or involuntary part-time work.
Wong (2019)	Increase in minimum wages in Ecuador in 2012.	✓		Finds that change in minimum wage raises wages but less for women (who were mainly domestic workers) who also experienced greater reduction in hours and worked more hours. The increase in the minimum wage may have resulted in reduced earnings for high income workers as employers compensated, paying more to low-income workers by paying less to high income earners.	High	Given that only short-run periods of panel data were available (two consecutive quarters followed by a gap of two consecutive quarters and two other consecutive quarters) the ability to adjust for long-term trends was limited. The analysis covered a period of significant economic growth and the impacts during economic contraction may be different.
Gudibande and Arun (2020)	Introduction of minimum wage legislation at local level in India (before national law was introduced in 2019).	✓	✓	Finds a positive short run effect on wages, less clear in the long run. No effect on the level of employment. Concludes that legislation alone cannot lead to any significant effects due to a large degree of non-enforcement. The laws do not require employers to register informal workers, making it difficult to monitor enforcement.	High	Studies focused on domestic employment have limited applicability to discussions regarding the impact of minimum wage policies. Given that households are not businesses, it is doubtful that decisions about increasing minimum wages and employing informal labor are the same as those made by profit-oriented business that have several options in response to changes in minimum wages, including raising prices. Additionally, the output of domestic work does not have a price that can be increased in response to an increase in the minimum wage.
Işlk et al. (2020)	Increase in minimum wages in Turkey in 2016.	✓	✓	Finds that wages went up for both formal and informal workers without impacting employment negatively. Women in lower education brackets experienced a smaller wage increase. Some increase in informal employment among those without university education.	High	Focuses only on the short-run effects of the minimum wage increase.
Nyirenda and Chibomba (2020)	Introduction of minimum wage policy in Zambia, first adopted in 1992.	✓	✓	Small positive impact on real wages in the short run but not in the long run. No impact on the extensive margin in terms of employment opportunities or the probability of being employed as domestic worker in the short and long run.	Medium	Small sample, consisting of only 30 observations, with a lot of weight put onto relatively minor changes in distribution. It is unclear if the finding for changes in length of contract was a measure of change in contract length as interpreted vs. a spot measure of contract length at one point in time.
Siregar (2020)	Increase in minimum wages in Indonesia in 2001.		✓	A raise in minimum wage reduces formal sector employment after a lag, with a higher effect for women than men, and increases informal employment after a lag. The change reduces unemployment but may decrease labor market participation.	High	A large informal sector, with only 42% of workers identified as being in the formal sector. The authors refer to enforcement of minimum wage regulations in Indonesia as being particularly problematic. Enforcement difficulties may, in turn, bias findings regarding implications of increases in minimum wages.
Katzkowicz et al. (2021)	Existing minimum wage policy in Uruguay, first introduced in 2006.	✓	✓	Finds that while wages in both formal and informal sectors went up by 20%, the employment rates in both declined. Two thirds earned less than the minimum wage, indicating significant noncompliance with the law. 15% exited the domestic work sector (not clear where they ended up) and two thirds of formal domestic workers migrated to the informal sector.	Medium	Studies focused on domestic employment have limited applicability to discussions regarding the impact of minimum wage policies. Given that households are not businesses, it is doubtful that decisions about increasing minimum wages and employing informal labor are the same as those made by profit-oriented business that have several options in response to changes in minimum wages, including raising prices. Additionally, the output of domestic work does not have a price that can be increased in response to an increase in the minimum wage.
Kyaw and Cho (2021)	Introduction of minimum wage policy in Myanmar 2015.		✓	Finds no negative effect of minimum wage on full-time employment but some sectoral shifts. Female employment increased and part-time employment decreased.	High	The analysis covers 3 years only and is at enterprise level, being limited to major industrial urban sectors only. Does not consider internal management issues and policy context.

*Quality appraisal rating interpretation: To calculate the rating, we used the number of “Yes” responses to the quality assessment questions included in the MMAT 2018 version, including the two screening questions. Low quality (0–2 “Yes” answers), medium quality (3–5 “Yes” answers), and high quality (6–7 “Yes” answers).

There were three types of initiatives evaluated: (*a*) newly introduced minimum wage policies (three studies); (*b*) existing minimum wage policies (three studies), and (*c*) increases in existing minimum wage policies (ten studies). The outcomes evaluated were: (*a*) workers’ financial compensation (including the ways in which regulations in the formal sector affect compensation of all workers in the formal sector, including the precariously employed, and the ways in which regulations in the formal sector affect wages for workers in the informal sector) and (*b*) employment security (the ways in which minimum wage policies in the formal sector can result in changes in workers’ employment arrangements, for example, status quo, formal/informal to unemployment, formal to informal, or informal to formal). Twelve studies examined changes to workers’ financial compensation, 14 focused on changes related to their employment security, and ten studies examined changes to both outcomes. While we acknowledge that formal employment is not always secure, we argue that informal employment, regardless of choice, is insecure. Although some people have more tolerance for insecurity, depending on family wealth, structures, or dynamics, we argue that an informal job is insecure even if it reflects a worker's choice.

### Study Results

Effects of minimum wage policies on workers’ financial compensation. The results of the 12 studies evaluating the effects of such policies on wages suggest that minimum wage policies could be linked to increased financial compensation, although with some variation. Since 10 of those studies were longitudinal, it is possible that the observed effects were the result of other factors unrelated to minimum wage policies, given that various contextual changes can affect a country's labor market and economy over extended periods. The positive effect on compensation was experienced by both formal and informal workers. Specifically, out of eight studies covering both formal and informal workers, three found general wage increases^61–63^; one found positive effects on formal sector wages but no spillover effects on the informal sector^64^; one found positive effects for formal sector workers and negative ones for informal workers^65^; one found positive effects for informal sector workers but not for the formal ones^66^; yet another one reported increased wage compensation only for workers earning close to the minimum wage .^41^ In another study, the focus was on effects on the overall wage gap between formal and informal workers instead of on effects on individual worker groups, suggesting that the gap stayed the same.^38^ Five of the 16 studies targeted specific economic sectors recognized for their high levels of precarious employment. One of those, which examined five sectors, found wage increases in the retail, domestic, and security sector but not in the forestry and taxi sectors.^67^ Three studied the domestic sector, where two found positive effects on wages,^47,63^ while the third found only a small positive effect in the short run and no effect in the long run.^68^ The fifth, which focused on agricultural and domestic workers, found an overall increase in wages that was larger among men than women, who were mainly represented in the domestic worker category.^69^ Overall, studies that found positive effects were longitudinal in design.

Effects of minimum wage policies on workers’ employment security. In summary, the 14 studies evaluating effects of minimum wages on employment showed little or no effects. Eleven studies resulted in variations of status quo. Seven of them reported no negative impact on total employment.^38,47,61,62,64,67,68^ One study resulted in reduced unemployment but possibly also a decrease in labor market participation,^70^ and one showed no negative effects on full-time employment, an increase in female employment, and a decrease in part-time employment.^71^ Three studies reported an increased probability of unemployment.^41,63,65^

Among the eight studies evaluating the effects of minimum wages on informal employment, four showed results indicating a decline in such employment, one was somewhere “in between,” and three indicated an increase. In the group of studies indicating a decline in informal sector employment, three also showed an increase in formal sector employment.^64,65,72^ Another study concluded there was no increase of informal employment^61^ while the one with “in between” findings showed that only very few formal workers moved to the informal sector.^38^ One of the three studies resulting in an increase in informal sector work indicated that the increase took place after a lag,^70^ which could possibly mean a link to context factors not accounted for. The second study in this category suggested that two thirds of formal domestic workers migrated to the informal sector^63^ while the third study pointed to a reduction in the probability of youth moving from informal to formal employment.^73^ Overall, studies that found positive effects had a longitudinal design. However, the follow-up for the study by Jimenez^72^ was limited to one year.

Several key methodological considerations are included in Table 3 to support the interpretation of the findings.

### Quality Appraisal

Table 4 displays the results of the MMAT quality assessment, including the detailed rating for each criterion. Overall, the ratings were very positive. Out of the 16 studies, 15 used quantitative descriptive methods and one used mixed methods. All the included studies had clear research questions and in all studies except one,^68^ the collected data allowed the authors to address the research questions.

**Table 4.** Critical Appraisal of the Included Articles.

	SCREENING QUESTIONS		4. QUANTITATIVE DESCRIPTIVE STUDIES				
Leading author and publication year	S1. Are there clear research questions?	S2. Do the collected data allow to address the research questions?	4.1. Is the sampling strategy relevant to address the research question?	4.2. Is the sample representative of the target population?	4.3. Are the measurements appropriate?	4.4. Is the risk of nonresponse bias low?	4.5. Is the statistical analysis appropriate to answer the research question?
Maloney and Mendez, 2004	Yes	Yes	Yes	Yes	Yes	Can’t tell	Yes
Bhorat, Kanbur, and Mayet, 2013	Yes	Yes	Yes	Yes	Yes	Yes	Can’t tell
Khamis, 2013	Yes	Yes	Yes	Yes	Yes	Yes	Yes
McCann et al., 2014 (Groisman 2014)	Yes	Yes	Yes	Yes	Yes	Yes	Yes
Aslan and Kirat, 2015	Yes	Yes	Yes	Yes	Yes	Yes	Yes
Hohberg and Lay, 2015	Yes	Yes	Yes	Yes	Yes	Yes	Yes
Groisman, 2016	Yes	Yes	Yes	Yes	Yes	Yes	Yes
Pratomo, 2016	Yes	Yes	Yes	Yes	Yes	Yes	Yes
Jimenez, 2018	Yes	Yes	Yes	Yes	Yes	Yes	Yes
Wong, 2019	Yes	Yes	Yes	Yes	Yes	Yes	Yes
Gudibande and Jacob, 2020	Yes	Yes	Yes	Yes	Yes	Yes	Yes
Işik, Orhangazi, and Tekgüç, 2020	Yes	Yes	Yes	Yes	Yes	Yes	Yes
Siregar, 2020	Yes	Yes	Yes	Yes	Yes	Yes	Yes
Katzkowicz, Pedetti, Querejeta, and Bergolo, 2021	Yes	Yes	Yes	Yes	Can’t tell	Yes	Can’t tell
Kyaw and Cho, 2021	Yes	Yes	Yes	Yes	Yes	Can’t tell	Yes
	SCREENING QUESTIONS		5. MIXED METHODS STUDIES				
Leading author and publication year	S1. Are there clear research questions?	S2. Do the collected data allow to address the research questions?	5.1. Is there an adequate rationale for using a mixed methods design to address the research question?	5.2. Are the different components of the study effectively integrated to answer the research question?	5.3. Are the outputs of the integration of qualitative and quantitative components adequately interpreted?	5.4. Are divergences and inconsistencies between quantitative and qualitative results adequately addressed?	5.5. Do the different components of the study adhere to the quality criteria of each tradition of the methods involved?
Nyirenda and Chibomba, 2020	Yes	No	Can’t tell	Yes	No	Can’t tell	Yes

Risk of bias assessment of included studies using the Mixed Methods Appraisal Tool (MMAT). This table uses the same categories and format of the MMAT tool [add citation]. The 16 studies are grouped into two categories according to study design: Quantitative descriptive studies × 15 and Mixed methods studies × 1. The quality assessment questions are slightly different for the two study designs but have the same answer options: Yes, No, and Can’t tell.

Among the quantitative descriptive studies, all had relevant sampling strategies to address the research question and a sample that was representative of the target population. The measurements were deemed appropriate except in one case^63^ where the reviewers could not tell. On the question about the risk of nonresponse being low, the assessment was positive for all studies except for Maloney and Mendez,^41^ in which case the reviewers could not tell. The assessment was similarly positive on the question whether the statistical analysis was appropriate to answer the research question, except for two studies in which cases the reviewers could not tell.^63,67^

The assessment of the mixed methods study^68^ was slightly less favorable than for the quantitative studies. It was not clear whether there was an adequate rationale for using a mixed method design to address the research question, nor if divergences and inconsistencies between quantitative and qualitative results were adequately addressed. Also, the outputs of the integration of qualitative and quantitative components were not adequately interpreted. On the other hand, the different components of the study were effectively integrated to answer the research question, and they adhered to the quality criteria of the traditions of these methods.

## Discussion

In this literature review of the impact of minimum wage policies on workers in precarious employment, we have included studies focusing on both formal and informal workers. This is based on the premise that a sufficient minimum wage is crucial for ensuring fair working conditions, protecting workers in low-paying jobs, including those in precarious and informal employment setups. The review focused on the impact of minimum wage policies on financial compensation and employment security: (*a*) for low-wage workers in the formal sector, who are likely to be precariously employed, and (*b*) for low-wage workers in the informal sector, who we also consider to be precariously employed since they lack a formal relationship with their employer. The results suggest that minimum wage policies could be linked to increased financial compensation for workers in precarious, including informal, employment arrangements, although with some variation. In turn, increased financial compensation is likely to mitigate exposure to precarious employment for workers in formal and informal work and for workers whose degree of precarity changes as they shift between low paid formal work and informal work. The impact of minimum wage policies on employment security ranged from no effects to neutral effects on full-time employment and to an increased probability of unemployment. Out of the 16 studies that met our inclusion criteria, none were from high-wage economies. Therefore, although the findings of these studies may have implications for higher-wage economies contexts, it is not currently feasible to extend these conclusions beyond low-wage economies.

There are several explanations regarding the lack of studies evaluating the effects of minimum wage policies on dimensions of precarious employment in high-wage countries and their potential role in moving workers into the informal sector. First, in high-wage economies, most employment, including most precarious employment, is found in the formal sector, governed by labor regulations such as minimum wage policies. Although it is difficult to estimate the size of the unregulated informal sector in high-wage economies, we know that it is smaller than in low-wage economies and dominated by higher income, self-employed professionals, and higher skilled, independent contractors.^74^ Until the recent spread of low-wage employment among unregulated digital platform companies in high-wage economies, labor regulations posed challenges for employers attempting to transition precariously employed workers from the formal to the informal sector. For instance, large retail chains that contract out tasks do so largely to firms that also employ workers in the formal sector.^25^ In contrast, the rise of digitization happening in the transportation sector made it easy for employed cab drivers to be converted into cheap-fair service drivers in the informal, unregulated sector. The studies we found concern only low-wage economies, which are commonly characterized by (*a*) fewer workers in standard employment relationships and smaller regulated formal sectors, in combination with (*b*) larger unregulated informal sectors, where most of the employment is low-wage and lacks a contractual agreement, which means that it can therefore be regarded as being the equivalent of precarious employment in the formal sector.

A significant aspect that affects the relationship between minimum wage policies and precarious employment is law reinforcement. A minimum wage law remains symbolic if it is not enforced, regardless of whether the setting is a low- or high-income country. In countries struggling with corruption or inadequate funding, enforcement is problematic. Further, where collective agreements are vital parts of the wage setting system, the weakening of workers’ and employers’ organizations pose another threat to enforcement.

Despite ongoing debate among academics and (mostly) politicians, the underlying assumption is often that employers in economies composed of specialized higher skilled workers and smaller informal sectors, such as those found in Europe and North America, will respond to a minimum wage increase by raising output prices or reduce total employment^75^ rather than push workers into informal employment. Further, most studies on the impact of minimum wage policies in higher wage economies predate the more recent focus of research on the issue of precarious employment, which partially explains the lack of focus on the impact of policy change on workers in precarious employment.

By contrast, we believe that in economies with large unregulated informal sectors, where much of the work is similar to work done by workers in precarious employment in the formal sector, employers have more options in response to policy changes. Consequently, employers can reduce formal employment (which could be both standard and precarious) in response to the increase in the minimum wage or they can avoid wage increases by moving employment from the regulated formal sector to the unregulated informal sector. However, even when the conclusion is that increased minimum wages decrease employment, without considering the number of hours worked, the overall decline in employment may be exaggerated given that job losses are mostly affecting low-paid, part-time workers.^76^ Commonly, studies of economies with large informal sectors focus on three questions: the impact on overall employment, the changing prevalence of formal and informal employment, and the impact of policy changes on wages in the informal sector, referred to as the “lighthouse effect.” As unregulated platform employment grows in high-wage economies, understanding the ways in which changes in formal sector regulations might impact these workers becomes increasingly relevant.

Given the very different structure of labor markets in higher and lower wage economies, what can be learned from the papers identified by our review? Unfortunately, there were very few evaluated studies that attempted to explore the impact of minimum wage regulation on workers in precarious, including informal, employment and those in sectors recognized for having high proportions of precarious employment, and each these studies was set in a low-wage economy. Further, there was little commonality in the details of study design and little description and/or overlap in the context of regulations operating in the different jurisdictions studied. Overall, the findings on the impact of minimum wage policies on the financial compensation of workers in precarious, including informal, employment arrangements suggest positive effects, however some of the findings were contradictory. For instance, for overall wage improvement, one study reported positive impacts for formal but not for informal workers,^64^ one found the reverse,^66^ and others found income increases for both groups,^41,61–63^ albeit possibly only close to the marginal wage. Similarly, for domestic workers, the most common specific sector, results were positive but possibly short-lived.^47,68^

There are several limitations to the studies reviewed. One is their origin in a relatively small subset of only 10 low-wage countries, with Argentina being the focus in four studies and Indonesia in three. The studies were relatively data-rich, all but one relying on data collected by national agencies. This suggests that data availability has determined the countries included in the study, raising questions about the applicability of the findings to other countries. Only a few of the studies examined the impact of employment changes in specific sectors or types of workers with different identity factors (e.g., gender, age, education level, ability, migration/citizenship status). Those that did reported findings among younger workers or female workers. This points to the need for more research on the ways in which changes in minimum wage regulations may affect worker subgroups differently. Also, the definitions of formal and informal workers differed significantly across studies, making the comparison of findings difficult. Further, while the quality of the studies was high, each one had several limitations that should be carefully considered when interpreting the results. A significant limitation was that most of the studies have a longitudinal component, which made it difficult to sort out the impact of changes in minimum wage policy from other contextual changes taking place simultaneously in the economy, such as economic growth or decline, broader policy changes, and changing trade relations. None of the studies was able to fully isolate changes in minimum wage regulations from other changes taking place, thus leaving open the possibility that the observed effects were in fact the result of factors unrelated to minimum wage policies. These studies contribute to an increased understanding of the impacts of minimum wage legislation on precarious, including informal, workers despite a range of study limitations and some contradictory findings, which we believe should be seen in the context of (*a*) scarce employment data due to a large informal sector; (*b*) underfunded governmental organizations; and (*c*) a less developed research infrastructure, all of which may influence low-income countries’ capacity to collect/access data, or monitor enforcement of minimum wage legislation.

Overall, due to the low number of relevant studies available to answer our research questions, along with the other challenges we refer to in this section, our review contributes only limited findings. Additionally, these findings are mostly relevant to low-income economies but not the high-income ones, which clearly highlights a research gap and the need for studies examining the impact of minimum wages on high-wage economies, which is especially important given the gradual expansion of informal and precarious work in these economies.

## Conclusion

The exposure of workers to precarious employment has complex health and well-being repercussions, giving rise to serious population health and health equity challenges. For this reason, a better understanding of strategies with the potential to address precarious employment is needed. This article presents available evidence from 16 studies that have assessed the effects of minimum wage policies on workers’ financial compensation and employment security. While we recognize that minimum wage policies do not purposefully address workers’ employment arrangements, representation, and nonmonetary rights and benefits, they have the potential to reduce or mitigate income inadequacy through increases in financial compensation, a key consideration given that a comprehensive review conducted recently revealed a dearth of evaluated interventions addressing precarious employment.

The 16 studies identified three types of initiatives: newly introduced minimum wage policies, existing minimum wage policies, and adjustments to existing minimum wage policies aimed at formal workers, including precarious workers, informal workers, or both. We included initiatives that focused on both formal and informal workers because precarious employment can exist in the formal as well as the informal sector and formal and informal workers share many concerns regarding income and would benefit from minimum wage policies. The evaluated outcomes focused on changes to workers’ financial compensation and employment security. While the evidence suggested that minimum wage policies could be linked to increased financial compensation, although with some variation, there was little or no effect on employment security.

Despite a large body of literature addressing minimum wage policies, only a handful of studies fitted our review's inclusion criteria, thus reflecting a limited focus on the impact of minimum wages on workers in precarious, including informal, employment in the literature. Notwithstanding these limitations, the identified studies call attention to the various ways in which minimum wage regulations might affect workers in economies with a high prevalence of informal and/or precarious employment. Additionally, these studies should alert higher wage economies to the need for related research and policy as their unregulated informal sectors grow. Such research should focus on the interaction between minimum wage policies and the distribution of employment between the formal and informal sectors as the informal sector expands. In addition, since researchers in higher wage economies generally have access to richer public data sources, they should regularly examine how policy changes affect the prevalence of precarious employment and terms of employment in both the formal and informal sector. The studies reviewed in this paper provide insights into how such research might be conducted. For instance, special consideration should be given to using common definitions of formal, informal, and precarious employment and describing or accounting for the overall sociopolitical context that may influence the effect of minimum wage policies. Additionally, efforts should be made to provide financial and infrastructure support for the systematic reporting of initiatives and evidence-informed practices across the world, which could be shared and implemented once consideration for similarities and differences in context have been given. Last, but not least, a research strategy should be adopted that focuses on evaluating the potential of various regulations, particularly minimum wage policies, to address precarious employment.

## Supplemental Material

sj-docx-1-joh-10.1177_27551938241286463 - Supplemental material for What is the Role of Minimum Wages in Addressing Precarious Employment in the Informal and Formal Sectors? Findings from a Systematic Review Supplemental material, sj-docx-1-joh-10.1177_27551938241286463 for What is the Role of Minimum Wages in Addressing Precarious Employment in the Informal and Formal Sectors? Findings from a Systematic Review by Carin Håkansta, Virginia Gunn, Bertina Kreshpaj, Nuria Matilla-Santander, David H. Wegman, Christer Hogstedt, Emilia F. Vignola, Carles Muntaner, Theo Bodin, Patricia O’Campo and Wayne Lewchuk in International Journal of Social Determinants of Health and Health Services
