# Supplementary material for: What is the Role of Minimum Wages in Addressing Precarious Employment in the Informal and Formal Sectors? Findings from a Systematic Review
Source: Int J Soc Determinants Health Health Serv. 2024 Oct 7;55(2):124–47. doi: 10.1177/27551938241286463 (PMC11977811; doi:10.1177/27551938241286463)
Supplement: sj-docx-1-joh-10.1177_27551938241286463 - Supplemental material for What is the Role of Minimum Wages in Addressing Precarious Employment in the Informal and Formal Sectors? Findings from a Systematic Review [file sj-docx-1-joh-10.1177_27551938241286463.docx]

| Study author(s)  Publication Year | Countries Examined/ Language | Type of Evidence | Population of Interest and Targeted economic sector | Design and data collection approaches used to evaluate initiatives | Study Objectives |
| --- | --- | --- | --- | --- | --- |
| Maloney and Mendez, (2004) | Latin America, with Colombia as a case/ English | Book chapter published by National Bureau of Economic Research | Formal and informal workers (no specific sector). | Quantitative descriptive study based on panel data from the National Housing Survey in Colombia since 1997 with a rotating sample of 25% of households. Uses numerical measures and kernel density plots to show the impact of minimum wage on the distribution of wages, with a focus on the informal sector and on higher wages. Tracks employment movement from having a job to becoming unemployed from one quarter to the next and not longer-term effects beyond three months. | To quantify the effects, at 3 months of raising the minimum wage on worker wages, and the move from employment to unemployment. |
| Bhorat et al (2013) | South Africa/ English | Academic journal article | Formal workers in sectors with high levels of precarious employment (retail, domestic work, forestry, security and taxi). | Quantitative descriptive study based on 15 waves of biannual Labour Force Survey data (2000 - 2007). Using a quasi-experimental approach, two alternative specifications of a difference-in-differences model were applied in order to estimate the impact of wage laws on employment, wages and hours of work in five sectors. A unique control group was established for each sector. | To assess the effects of minimum wage laws on employment, wages, and  hours of work in five sectors. |
| Khamis (2013) | Argentina/ English | Academic journal article | Formal and informal workers (no specific sector). | Quantitative descriptive study based on Argentine National Household data 1993-2004. As control group, the paper exploits the different prevalence of low wage workers in regions of Argentina and assumes that regions with few low wage workers will be less affected by changes in minimum wage and can be used as the control. | To assess the effects of minimum wage on wages. |
| Groisman (2014) | Latin America, with Argentina as a case / English | Book chapter published by International Labour Organization. | Formal and informal workers (no specific sector). | Quantitative descriptive study based mainly on pooled panel data (14,000 households) 2004-2013 and a simple distribution of minimum wage workers. It uses multinomial logistic regression to assess the effects of minimum wage on employment, informality and wages. | To assess the effects of minimum wage on employment, informality, and wages. |
| Aslan and Kirat (2015) | Turkey/ French | Academic journal article | Formal and informal workers (no specific sector). | Quantitative descriptive study based on aggregate data from 26 annual observations collected by the Turkish National Statistics Institute (1988-2013). Regresses level of employment on minimum wage, level of economic activity, level of active population. | To assess the effects of minimum wage on wages. |
| Hohberg and Lay (2015) | Indonesia/ English | Academic journal article | Formal and informal workers (no specific sector). | Quantitative descriptive study based on data on the self-employed (48,030 observations and 18,825 individuals) in Indonesia 1997-2007 from Indonesian Family Life Survey, three waves. The unit of analysis is the individual. Fixed effects regression at the individual level to assess the effects of minimum wage on informal and formal sector wages and employment. | To assess the effects of minimum wage on employment, informality, and wages. |
| Groisman (2016) | Argentina/ English | Book chapter in Research on Economic Inequality. | Formal and informal workers (no specific sector). | Quantitative descriptive study based on longitudinal, individual annual panel data (2004-2013) from Argentina National Institute of Statistics Permanent Household Survey, using multinomial logistic regression models. The analysis examines if people earning near the minimum wage lose jobs when the minimum wage changes. | To assess the effects of minimum wage and income transfer programmes on the economic participation of the population and the informal sector. |
| Pratomo (2016) | Indonesia/ English | Academic journal article | Formal and informal workers (youth) (no specific sector) | Quantitative descriptive study based on the National Labour Force Survey (2010-2012); a logit model is used to examine the distribution of youth employment across five different categories of the employment relationship when the minimum wage changes. The unit of analysis is the individual. The model includes separate analyses for urban and rural areas and for gender. | To assess the effects of minimum wage on youth with different employment status 2010 - 2012. |
| Jimenez (2018) | Argentina/ Spanish | Academic journal article | Formal and informal workers (no specific sector). | Quantitative descriptive study based on data from the Argentine Permanent Household Survey for the period 2004-2005, a difference in difference approach is used to examine a) regional variety in prevalence of workers earning near or below the minimum wage and b) median wage within urban regions of Argentina. | To assess the effects of a minimum wage increase in 2004-2005 on transitions to quality jobs. |
| Wong (2019) | Ecuador/ English | Academic journal article | Formal low-income workers including workers in sectors with high levels of precarious employment (domestic work and agriculture). | Quantitative descriptive study based on individual panel data drawn from a household panel in 2012 (National Institute of Statistics and Census), the difference in difference approach was used to analyze the effects on hours worked and wages among low-wage workers of the 2012 minimum wage increase. | To assess the effects of a minimum wage increase on wages and hours worked among low-wage workers in Ecuador. |
| Gudibande and Arun (2020) | India/ English | Academic journal article | Informal workers (including maid servants, cooks and watch men). | Quantitative descriptive study based on cross-sectional household data from a National Sample Survey in India (2004-2012). Units of analysis are the state and the individual in different states. Exploits the fact that not all states have a minimum wage for domestic workers. Uses matched pairs in treatment/non-treatment states (in total 16). | To assess the impact of minimum wage legislation on wages and employment opportunities. |
| Işlk et al. (2020) | Turkey/ English | Academic journal article | Formal and informal workers (no specific sector). | Quantitative descriptive study based on data from the annual Household Labor Force Survey (2009-2016). Unit analysis is aggregate data on key sectors of the workforce (age, education, formal, informal). Individual data is used to assess outcomes by controlling for the % of workers in a region affected by the change in minimum wage. Uses simple before and after comparisons of prevalence of workers below or near minimum wage and regression analysis to study regional effects of a national minimum wage. | To assess the effects of a minimum wage increase on wages, informality, and employment. |
| Nyirenda and Chibomba (2020) | Zambia, with the City of Chipata as a case/ English | Academic journal article | Formal workers in sectors with high levels of precarious employment (domestic work). | Mixed methods study based on a descriptive research design and purposive sampling, using researcher collected surveys and interviews with employers and domestic workers in 30 households in 2018. It also uses secondary data from textbooks, journals and other on-line resources. A combination of thematic and statistical analyses was used. | To assess the effects of minimum wage policy on wages and employment opportunities. |
| Siregar (2020) | Indonesia/ English | Academic journal article | Formal and informal workers (no specific sector). | Quantitative descriptive study based on aggregate panel data covering 26 provinces in Indonesia, collected from the Indonesia Labor Force Survey (2001-2015) and the Ministry of Manpower and Transmigration. It uses a dynamic panel data estimation method, specifically the general methods of moments estimator to correct for error term problems. | To assess the impact of minimum wage on employment, formal and informal employment. |
| Katzkowicz et al. (2021) | Uruguay/ English | Academic journal article | Formal and informal workers (domestic work). | Quantitative descriptive study based on cross sectional pooled data from National Household Survey in Uruguay (2006-2016) using a dual-economy density-discontinuity design with latent values that would have existed without a minimum wage policy. Estimating the impact of minimum wages on unemployment, wages, and mobility of women domestic workers between the formal and informal sectors. | To assess the impact of minimum wage on wages, unemployment, and formal-informal sector mobility among women. |
| Kyaw and Cho (2021) | Myanmar/ English | Academic journal article | Formal and informal workers (no specific sector). | Quantitative descriptive study based on panel data from the World Bank Enterprise Survey across five major industrial sectors (in 2014 and 2016) using fixed-effects regression to estimate whether enterprises substitute machines for labour due to the rise of the minimum wage. | To assess the effects of minimum wage policy on full-time and part-time employment. |
